# Development of a Bioactive Carboxymethyl Cellulose (CMC)/Keratin/Platelet‐Rich Fibrin (PRF) Composite Scaffold for Periodontal Regeneration

**DOI:** 10.1155/bmri/9227735

**Published:** 2026-10-26

**Authors:** Saba Jaffarkhah, Raziyeh Najafloo, Saeedeh Zare Jalise, Mina Jarrahi, Esmaeil Biazar, Arezou Mehrabi

**Affiliations:** ^1^ Department of Biomedical Engineering To.C Islamic Azad University Tonekabon Iran tiau.ac.ir; ^2^ Department of Biomedical Engineering TeMS.C Islamic Azad University Tehran Iran tiau.ac.ir; ^3^ Department of Tissue Engineering and Applied Cell Sciences School of Medicine Qom University of Medical Sciences Qom Iran muq.ac.ir; ^4^ Department of Medicine To.C Islamic Azad University Tonekabon Iran tiau.ac.ir

**Keywords:** carboxymethyl cellulose, composite scaffold, keratin, periodontal regeneration, periodontitis, platelet-rich fibrin, tissue engineering

## Abstract

A chronic inflammatory condition, periodontitis leads to the gradual breakdown of the structures that anchor the teeth and represents a significant clinical problem in advanced stages. Periodontal tissue engineering has been proposed as an effective regenerative approach, combining biomaterial scaffolds with bioactive agents to restore damaged tissues. This study developed and evaluated a novel carboxymethyl cellulose (CMC)/keratin/platelet‐rich fibrin (PRF) composite scaffold for periodontal tissue regeneration. Porous CMC/keratin scaffolds with varying weight ratios were produced via freeze‐drying and chemically cross‐linked using glutaraldehyde (GLU). Following structural and mechanical assessment, the optimized CMC/keratin formulation (40/60) was selected and subsequently incorporated with PRF to improve biological performance. The resulting scaffolds were evaluated for chemical composition (FTIR), morphology (SEM), mechanical behavior, cytocompatibility (MTT assay), and cell adhesion using L929 fibroblast cells. FTIR spectroscopy suggested the presence of intermolecular interactions among CMC, keratin, and PRF within the composite scaffold. SEM analysis revealed a highly interconnected porous architecture in the optimized scaffold, which was preserved after PRF incorporation. PRF incorporation improved the mechanical performance of the scaffold, as evidenced by increases in tensile strength, elastic modulus, and toughness, whereas elongation at break remained almost unchanged. In vitro cytocompatibility assays demonstrated that all scaffolds were noncytotoxic, with the CMC/keratin/PRF composite exhibiting significantly higher cell viability compared with CMC/keratin scaffolds, as determined by the MTT assay. Qualitative SEM observations also suggested enhanced cell attachment and spreading on the PRF‐containing composite scaffold. Overall, the findings demonstrate that the synergistic integration of CMC, keratin, and PRF produces a bioactive scaffold with suitable structural, mechanical, and biological characteristics. The CMC/keratin/PRF composite scaffold shows suitable potential for periodontal tissue engineering; however, future studies involving periodontal‐specific cells and in vivo models are warranted to further validate its clinical applicability.

## 1. Introduction

Periodontitis is a persistent inflammatory disease that results in the gradual degradation of the periodontium—the tissues that support the teeth, such as the gums, periodontal ligament (PDL), cementum, and jawbone [1]. If left unmanaged, this multifactorial disease can lead to increased tooth mobility and eventual tooth loss [2]. Its pathogenesis involves a complex interaction between pathogenic biofilms and dysregulated host immune responses, further influenced by systemic and environmental factors such as diabetes mellitus and smoking [3]. Despite improvements in periodontal therapies and disease classification, predictable regeneration of lost periodontal tissues in advanced stages remains a considerable clinical challenge [4].

Standard therapeutic approaches for periodontitis, such as scaling and root planing, are effective in controlling inflammation and slowing disease progression in the early stages of periodontitis [5]. Nevertheless, in cases involving extensive tissue loss, regenerative surgical interventions are necessary to reestablish both the structure and functional restoration of the periodontium [6]. Traditional regenerative approaches, including bone grafting and guided tissue regeneration (GTR) membranes, have shown clinical efficacy but are associated with several limitations, such as restricted availability of autologous grafts, donor‐site morbidity, potential for infection or immune reactions, and inconsistent long‐term outcomes [7, 8]. These challenges have spurred increasing interest in periodontal tissue engineering as a promising alternative for predictable tissue regeneration [9].

Periodontal tissue engineering seeks to restore a fully functional periodontium by integrating biomaterial scaffolds, bioactive signaling molecules, and regenerative cells in a synergistic manner [10]. Among these elements, scaffolds play a central role by establishing a three‐dimensional (3D) microenvironment that facilitates cell adhesion, proliferation, migration, and differentiation [11, 12]. Unlike passive barrier membranes, bioactive scaffolds can actively influence cellular activities and promote the regenerative process by emulating the biochemical composition and structural architecture of the native extracellular matrix (ECM) [11, 13]. Consequently, careful selection and rational design of scaffold materials are essential for achieving effective periodontal regeneration [14].

The water‐soluble cellulose derivative carboxymethyl cellulose (CMC) has attracted significant attention in tissue engineering applications due to its inherent biocompatibility, biodegradability, hydrophilic nature, and ease of processing [15, 16]. Scaffolds based on CMC demonstrate favorable physicochemical characteristics and can form highly porous architectures suitable for tissue engineering [17]. Nevertheless, the inherent bioactivity and cell‐adhesive properties of pure CMC are limited, restricting its capacity to fully support cellular functions, which necessitates its modification or combination with bioactive agents [18, 19]. Despite its excellent biocompatibility and hydrophilicity, CMC exhibits relatively poor mechanical stability and limited cell‐interactive properties, necessitating combination with bioactive polymers [20].

The cysteine‐rich structural protein keratin, derived from abundant human sources such as hair and skin, has shown considerable promise for use in tissue engineering biomaterials [21]. Human‐derived keratin exhibits excellent biocompatibility, biodegradability, low immunogenicity, and intrinsic bioactivity [22]. Notably, as a structural protein, keratin possesses intrinsic cell‐binding domains, including arginine–glycine–aspartic acid (RGD), leucine–aspartic acid–valine (LDV), and glutamic acid–aspartic acid–serine (EDS), which are also conserved in native ECM proteins such as collagen and fibronectin [22]. These motifs enhance cell–matrix interactions, promoting adhesion, spreading, and proliferation. Evidence suggests that keratin‐based substrates can markedly improve fibroblast attachment and accelerate wound healing, highlighting its potential to augment the biological performance of polysaccharide‐based scaffolds like CMC [23–25]. Keratin was incorporated to improve cellular interactions through its intrinsic cell‐binding motifs while enhancing the biological performance of the CMC‐based scaffold.

In addition to scaffold composition, the incorporation of bioactive signaling molecules is essential for guiding cellular behavior and promoting tissue regeneration [26, 27]. Platelet‐rich fibrin (PRF) is an autologous blood‐derived biomaterial composed of a dense fibrin matrix enriched with platelets, leukocytes, and a variety of growth factors, including platelet‐derived growth factor (PDGF), transforming growth factor‐*β* (TGF‐*β*), and vascular endothelial growth factor (VEGF) [28]. PRF has been extensively applied in periodontal and maxillofacial therapies due to its capacity to enhance angiogenesis, cell proliferation, and tissue repair [29, 30]. Compared with platelet‐rich plasma (PRP), PRF does not require anticoagulants and provides a sustained release of growth factors, rendering it particularly advantageous for tissue engineering applications [31]. Unlike conventional PRF membranes that are applied directly to the defect site, incorporation of lyophilized PRF into a porous scaffold provides a structurally stable platform that may prolong the local bioactivity of PRF while supporting cell infiltration and tissue regeneration. In addition to serving as a reservoir of growth factors, PRF exerts immunomodulatory and paracrine effects that contribute to tissue repair and regeneration [32]. Recent evidence indicates that PRF‐derived extracellular vesicles may function as paracrine mediators involved in immune modulation and regenerative signaling in periodontal tissues [33].

Therefore, the objective of this work is to fabricate a novel CMC/keratin/PRF composite scaffold and assess its potential as a bioactive platform for periodontal tissue engineering. We hypothesize that integrating CMC as a structural matrix, keratin as a bioactive ECM‐mimicking component, and PRF as a source of endogenous growth factors will synergistically improve the scaffold′s physicochemical properties, mechanical performance, and biological activity. To evaluate this hypothesis, CMC/keratin scaffolds with varying compositions were fabricated and characterized, and the optimized formulation was subsequently enriched with PRF. The resulting scaffolds were systematically assessed in terms of their structural, mechanical, and in vitro biological properties to determine their suitability as a promising scaffold for periodontal tissue engineering.

## 2. Materials and Methods

### 2.1. Preparation of PRF

This study was conducted in accordance with the Declaration of Helsinki and approved by the Institutional Research Ethics Committee (Approval No. IR.IAU.TON.REC.1404.152). Written informed consent was obtained from all volunteers prior to blood collection. Venous blood was collected from a single healthy, nonsmoking volunteer (male, aged 45 years) with no history of systemic disease or medication use within the preceding month. Approximately 10 mL of venous blood was collected per tube into sterile, glass‐coated, anticoagulant‐free tubes (Vacuette, Greiner Bio‐One, Austria). PRF was prepared according to the standardized Choukroun′s protocol. To ensure consistency, all tubes were processed within 2 min of collection. Blood samples were immediately centrifuged at room temperature using a fixed‐angle rotor centrifuge (Eppendorf 5810 R, Germany) at 400 × g (relative centrifugal force; RCF = 1680 rpm) for 12 min. After centrifugation, the PRF clot formed in the intermediate layer was carefully isolated from the underlying red blood cell fraction and the overlying platelet‐poor plasma using sterile surgical scissors and forceps. The harvested PRF clots were immediately frozen at −80°C for 24 h and subsequently lyophilized (Christ Alpha 1‐2 LDplus, Germany) at −55°C under a vacuum pressure of 0.1 mbar for 48 h. The freeze‐dried PRF was then mechanically milled using a cryogenic ball mill (Retsch MM400, Germany) at 30 Hz for 5 min. The resulting powder was sieved through a 100‐*μ*m mesh (Retsch, Germany) to obtain a uniform particle size of < 100 *μ*m, which was used for incorporation into the scaffold matrix. To ensure the quality and consistency of the PRF preparation, a peripheral blood sample was collected in an EDTA‐containing tube prior to centrifugation to determine the baseline platelet and leukocyte counts using an automated hematology analyzer (Sysmex KX‐21N, Japan), which revealed counts of 275 × 10^3^/*μ*L and 6.8 × 10^3^/*μ*L, respectively.

### 2.2. Fabrication of CMC/Keratin/PRF Composite Scaffolds

CMC (Sinopharm, China, MW = 80,000, DS = 0.8) was dissolved in deionized water under continuous stirring to obtain a homogeneous polymer solution. Keratin powder (Merck) was subsequently added in a stepwise manner at different weight ratios (CMC/keratin: 95/5, 90/10, 80/20, and 40/60) to evaluate the influence of keratin content on scaffold properties. The resulting mixtures were thoroughly homogenized to ensure uniform dispersion of keratin within the CMC matrix. Chemical cross‐linking was performed using glutaraldehyde (GLU, 0.05% w/v; Sigma‐Aldrich, United States) as the cross‐linking agent. The cross‐linking reaction was allowed to proceed for 4 h at room temperature under gentle stirring. To remove residual unreacted GLU and reduce potential cytotoxicity, the cross‐linked scaffolds were extensively washed with deionized water for 48 h at room temperature, with the water being replaced every 8 h. Based on physicochemical and structural analyses, the CMC/keratin scaffold with the optimal composition was selected for subsequent modification. To prepare the bioactive composite scaffold, PRF powder was incorporated into the optimized CMC/keratin solution at 10% (w/w) relative to the total polymer content. The mixture was then subjected to the same cross‐linking and freeze‐drying procedures described above. The fabricated scaffolds were designated according to their compositions, as summarized in Table 1.

**Table 1:** Composition and sample designations of the fabricated scaffold groups.

Sample name	CMC/keratin(w/w)	PRF
CMC/KRT 95/5	95/5	—
CMC/KRT 90/10	90/10	—
CMC/KRT 80/20	80/20	—
CMC/KRT 40/60	40/60	—
CMC/KRT/PRF 40/60	40/60	10% (w/w)

Prior to cell culture experiments, all fabricated scaffolds (after extensive washing) were sterilized by immersion in 70% ethanol for 2 h, followed by three successive washes with sterile phosphate‐buffered saline (PBS) for 30 min each. Finally, the scaffolds were exposed to UV irradiation (254 nm) for 1 h on each side under a laminar flow hood.

### 2.3. Scaffolds Characterization

#### 2.3.1. Fourier Transform Infrared Spectroscopy (FTIR)

FTIR analysis was performed to investigate the chemical structure and interactions among CMC, keratin, and PRF. FTIR spectra of raw PRF powder, CMC/keratin scaffolds, and CMC/keratin/PRF composite scaffolds were recorded using a Thermo Scientific Nicolet FTIR spectrometer (United States) in the range of 500–4000 cm^−1^ with a resolution of 4 cm^−1^. Sample preparation employed the standard KBr pellet method.

#### 2.3.2. Scanning Electron Microscope (SEM)

Scaffold morphology, including surface features and porosity, was examined using a SEM (XL30, Philips, The Netherlands) operated at an accelerating voltage of 15 kV. Scaffolds were cut into square specimens (approximately 1.5 cm^2^), sputter‐coated with a thin layer of gold, and observed at various magnifications to evaluate pore structure and interconnectivity.

#### 2.3.3. Mechanical Characterization

A universal testing machine (SANTAM; STM20) was employed to evaluate the mechanical performance of the scaffolds. Rectangular specimens measuring 30 × 10 × 1 mm were prepared from each scaffold formulation. For each formulation, five independently fabricated scaffolds were tested (*n* = 5 biological replicates). From each scaffold, three technical replicate specimens were prepared and tested to ensure reproducibility. Uniaxial tensile testing was performed at room temperature with a crosshead speed of 1 mm/min. Stress–strain curves were recorded, and tensile strength, elastic modulus, elongation at break, and toughness were calculated accordingly. All data are presented as mean ± SD (*n* = 5 independent scaffolds).

### 2.4. In Vitro Study

#### 2.4.1. MTT Assay

The in vitro cytocompatibility of the prepared scaffolds was assessed using an extract‐based MTT analysis [34]. Mouse fibroblast L929 cells (National Cell Bank, Pasteur Institute, Iran) were cultured in RPMI medium supplemented with 10% (v/v) fetal bovine serum (FBS; Invitrogen) and 1% penicillin–streptomycin (Lonza, Korea). Cells were seeded at a density of 3 × 10^4^ cells per well in 96‐well culture plates and maintained at 37°C in a humidified incubator containing 5% CO_2_. Scaffold extracts were prepared according to ISO 10993‐5 guidelines. Briefly, sterilized scaffolds were immersed in serum‐free RPMI medium at a concentration of 1 cm^2^/mL (surface area‐to‐medium volume ratio) and incubated at 37°C for 24 h under gentle shaking. The supernatant was then collected, filtered through a 0.22‐*μ*m syringe filter, and supplemented with 10% FBS and 1% penicillin–streptomycin immediately prior to use. For the PRF‐only control, lyophilized PRF was extracted at a concentration of 1 mg/mL, corresponding to the PRF content incorporated into the composite scaffold. Unlike the scaffold groups, which were extracted using a surface area‐to‐medium volume ratio of 1 cm^2^/mL, the PRF‐only group was extracted using a weight‐to‐volume ratio, owing to the absence of a defined scaffold surface area for this control. The PRF extract was incubated at 37°C for 24 h under gentle shaking and subsequently processed under identical filtration and supplementation conditions as described above for the scaffold extracts. Cells were treated with 100 *μ*L of the respective scaffold or PRF extracts and incubated for 3 and 7 days. At each time point, the culture medium was replaced with MTT solution (Sigma; 5 mg/mL in PBS), and cells were incubated for 3 h. The resulting formazan crystals were dissolved in dimethyl sulfoxide (DMSO; Merck), and optical absorbance was measured at 570 nm using a microplate reader (ELX808, BioTek). Untreated cells cultured on tissue culture plastic served as the negative control. All experiments were performed with three independent biological replicates (*n* = 3), with three technical replicates measured per independent experiment for each group and time point.

#### 2.4.2. Cell Attachment and Morphology Analysis on Scaffold

Cell attachment and morphology on the scaffold surfaces were evaluated using SEM [34]. The scaffolds were seeded with L929 fibroblasts and subsequently cultured for a period of 48 h. After incubation, the samples were gently rinsed with PBS, fixed with 2.5% GLU, and dehydrated through a graded ethanol series. The samples were then sputter‐coated with gold and observed by SEM to assess cell adhesion, spreading, and morphological features. Three independent experiments were performed for each scaffold group, and representative images are presented.

### 2.5. Statistical Analysis

Data are presented as mean ± SD. All experiments were performed with at least three independent biological replicates (*n* ≥ 3), with the specific *n* values indicated for each assay. Technical replicates were performed for each independent experiment as specified in the respective methods sections. Normality of data distribution was assessed using the Shapiro–Wilk test, and homogeneity of variances was evaluated using Levene′s test. For mechanical characterization data (comparison of two independent groups), an independent Student′s *t*‐test was applied. For the MTT assay (multiple groups), one‐way ANOVA followed by Tukey′s post hoc test was used. Statistically significant differences between groups are indicated in the corresponding table using an asterisk ( ^∗^), as defined in the table footnote. Statistical significance was set at *p* < 0.05. Analyses were performed using GraphPad Prism (Version 8; GraphPad Software, United States).

## 3. Results

### 3.1. Scaffolds Characterization

To elucidate chemical interactions and structural integration, FTIR spectroscopy was employed to characterize the CMC/keratin/PRF composite scaffolds (Figure 1). The FTIR spectrum of freeze‐dried PRF exhibited characteristic absorption bands associated with protein components. The prominent peak observed at approximately 1636 cm^−1^ corresponds to the Amide I band (C=O stretching), whereas the band near 1540 cm^−1^ is attributed to Amide II (N–H bending and C–N stretching). Additionally, the absorption band around 1238 cm^−1^ is assigned to Amide III vibrations, confirming the protein‐rich nature of the PRF matrix, which is mainly composed of fibrin.

**Figure 1:**
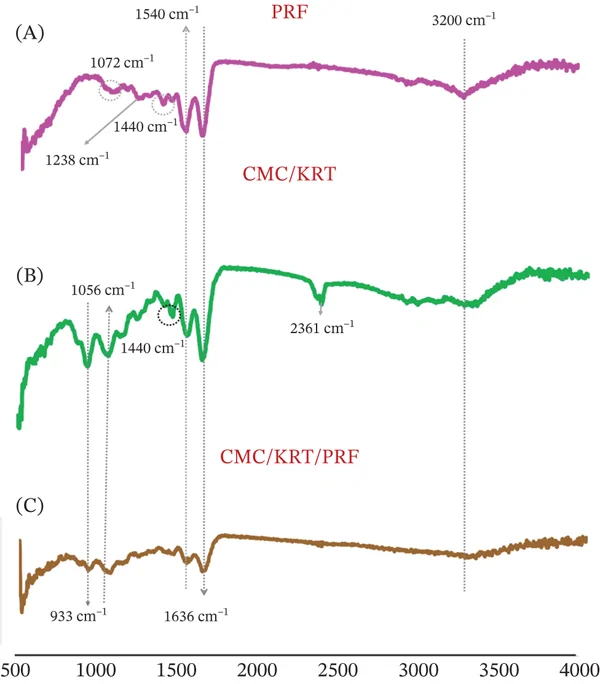
FTIR spectra of (A) lyophilized PRF powder, (B) CMC/keratin (40/60) scaffold, and (C) CMC/keratin/PRF composite scaffold.

The FTIR spectra of CMC/keratin scaffolds revealed signature peaks corresponding to both components. The peaks characteristic at approximately 1056 cm^−1^ are attributed to the C–O–C stretching vibrations of the polysaccharide backbone of CMC, whereas the band near 1440 cm^−1^ is associated with the symmetric stretching of carboxylate (COO^−^) groups. The presence of keratin was confirmed by the appearance of Amide I and Amide II bands at approximately 1636 and 1540 cm^−1^, respectively.

In the CMC/keratin/PRF composite scaffold, the Amide I band at around 1636 cm^−1^ remained detectable, although with reduced intensity. The emergence of an additional band around 933 cm^−1^ further suggests the formation of a cross‐linked network resulting from interactions among CMC, keratin, and PRF.

The surface morphology and porous architecture of the prepared scaffolds were examined using SEM (Figure 2). The freeze‐dried PRF powder exhibited an irregular, fibrillar morphology typical of lyophilized protein‐based materials. CMC/keratin scaffolds with lower keratin content (95/5 and 80/20) displayed a relatively heterogeneous pore structure with less uniform pore distribution and limited interconnectivity. In these samples, the scaffold walls appeared thinner and more fragile, which may negatively affect mechanical stability and cell infiltration. In contrast, the CMC/keratin (40/60) scaffold appeared to exhibit a porous structure with visually more interconnected pores and a relatively more uniform pore distribution compared with the other formulations. Based on these morphological observations, the CMC/keratin (40/60) scaffold was selected as the optimized formulation. Incorporation of PRF into this optimized scaffold did not visibly alter the porous architecture but led to a slightly rougher surface morphology.

**Figure 2:**
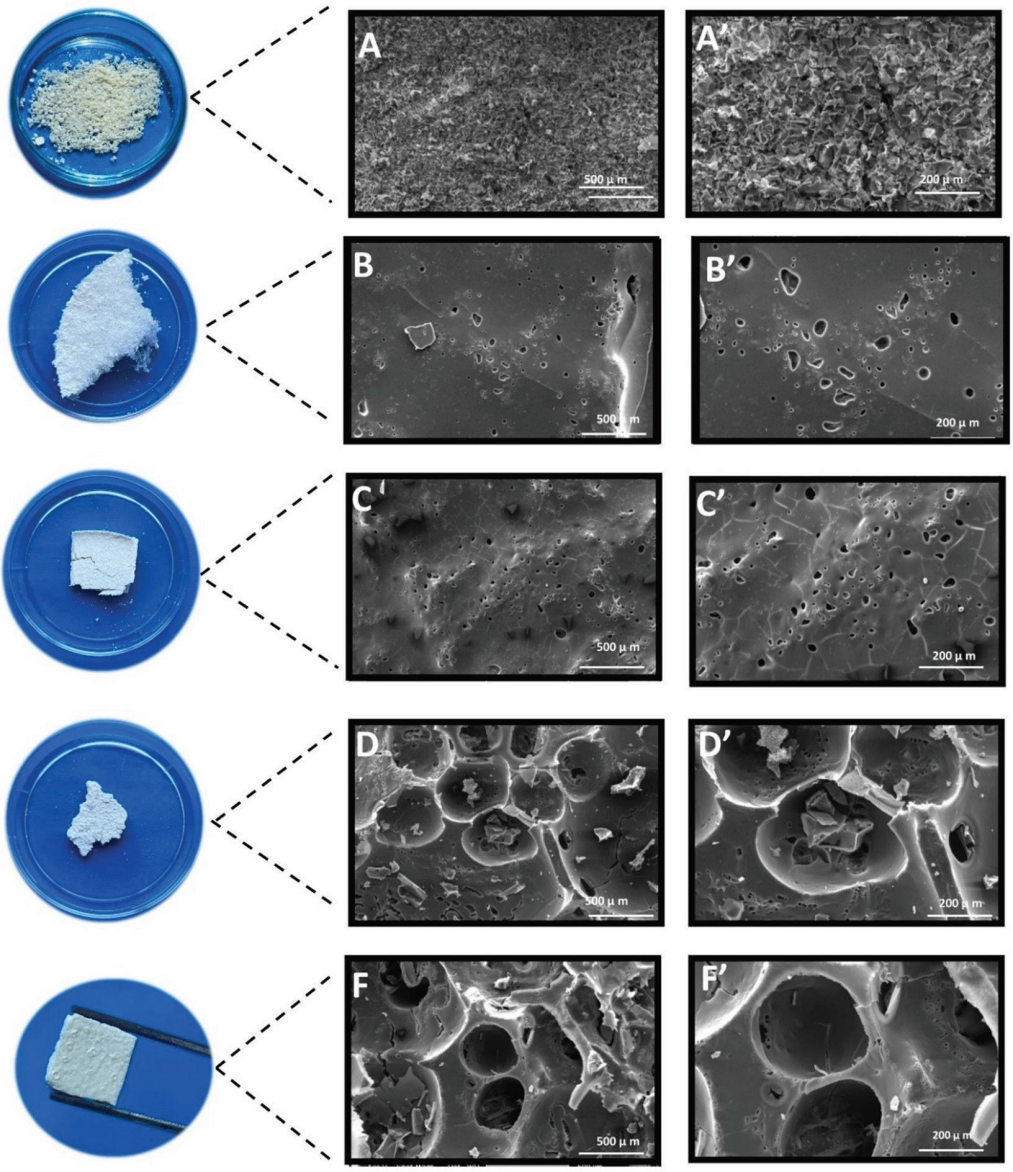
SEM micrographs of the prepared samples: (A,A′) lyophilized PRF powder; (B,B′) CMC/KRT (95/5) scaffold; (C,C′) CMC/KRT (80/20) scaffold; (D,D′) CMC/KRT (40/60) scaffold; and (F,F′) CMC/KRT/PRF composite scaffold prepared using the selected CMC/KRT (40/60) formulation.

### 3.2. Mechanical Properties

The stress–strain analysis was carried out to analyze the mechanical behavior of CMC/keratin (40/60) and CMC/keratin/PRF composite scaffolds (Figure 3), and the quantitative mechanical parameters are given in Table 2. PRF incorporation into the CMC/keratin (40/60) scaffold resulted in improved mechanical properties, with tensile strength increasing from 0.10 ± 0.02 to 0.13 ± 0.01 MPa and elastic modulus increasing from 0.60 ± 0.04 to 0.76 ± 0.03 MPa. Elongation at break remained almost unchanged (0.5% for both groups). Moreover, the PRF‐containing scaffold showed improved toughness (0.0488 ± 0.001 MJ/m^3^) compared with the CMC/keratin scaffold (0.0372 ± 0.002 MJ/m^3^), indicating its superior ability to absorb mechanical energy before fracture. All values are presented as mean ± SD (*n* = 5 independent scaffolds).

**Figure 3:**
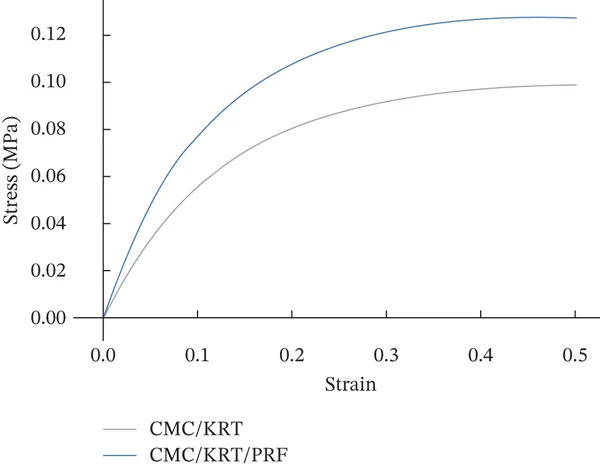
Stress–strain curves of the prepared composite scaffolds. The CMC/KRT (40/60) composite (Sample D) exhibited the highest mechanical strength among the prepared samples and was therefore selected as the optimized formulation. Incorporation of PRF into this composite (Sample F) resulted in improved mechanical properties, including increased tensile strength, elastic modulus (stiffness), and toughness, whereas elongation at break remained almost unchanged compared with the CMC/keratin (40/60) scaffold.

**Table 2:** Mechanical properties of the fabricated scaffold formulations (mean ± SD, *n* = 5).

Sample	Tensile strength (MPa)	Elastic modulus (MPa)	Elongation at break (%)	Toughness (MJ/m^3^)
CMC/KRT 40/60	0.1 ± 0.02	0. 6 ± 0.04	0.5 ± 0.01	0.0372 ± 0.002
CMC/KRT/PRF	0.13 ± 0.01 ^∗^	0.76 ± 0.03 ^∗^	0.5 ± 0.02	0.0488 ± 0.001 ^∗^

^∗^Significantly different from CMC/KRT 40/60 (independent Student′s *t*‐test, *p* < 0.05). Elongation at break: *p* > 0.05.

### 3.3. Cytotoxicity and Morphology Assessments

The cytocompatibility of PRF, CMC/keratin, and CMC/keratin/PRF scaffolds was evaluated using an extract‐based MTT assay with L929 fibroblast cells (Figure 4). The optical density values obtained from the assay reflect the metabolic activity and viability of cells exposed to scaffold extracts.

**Figure 4:**
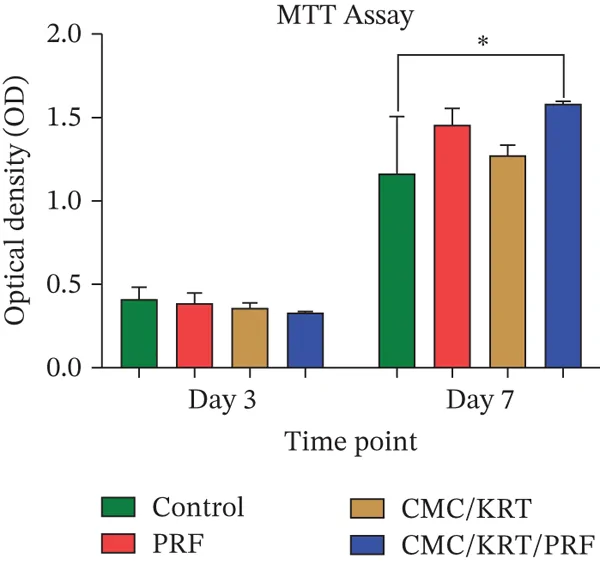
Cell viability of L929 fibroblasts cultured with extracts of PRF, CMC/keratin, and CMC/keratin/PRF scaffolds after 3 and 7 days, as determined by the MTT assay. Data are presented as mean ± SD (*n* = 3). Statistical analysis was performed using one‐way ANOVA followed by Tukey′s multiple comparisons test.  ^∗^ *p* < 0.05 compared with the control group.

After 3 days of incubation, all scaffold groups exhibited cell viability comparable with or higher than the control group, indicating the absence of cytotoxic effects. Notably, the CMC/keratin/PRF composite scaffold showed the highest cell viability among all tested groups, suggesting enhanced cytocompatibility during the early culture period. The CMC/keratin scaffold also supported cell viability, although to a lesser extent than the PRF‐containing composite.

By Day 7, a significant increase in cell metabolic activity was observed across all scaffold groups. The CMC/keratin/PRF scaffold maintained its superior performance, demonstrating significantly higher cell viability than the CMC/keratin scaffold and the control group. The PRF‐only group also showed a notable increase in cell viability over time, further supporting its bioactive role. Overall, these results demonstrate that all prepared scaffolds are cytocompatible, with the CMC/keratin/PRF composite exhibiting the highest cell viability among the tested groups.

SEM observations were used to evaluate cell attachment, spreading, and morphology on the scaffold surfaces after 48 h of culture (Figure 5). On the CMC/keratin scaffold, cells adhered to the surface but generally exhibited a rounded or slightly elongated morphology, with limited spreading and fewer cytoplasmic extensions. In contrast, cells cultured on the CMC/keratin/PRF composite scaffold displayed a well‐spread, spindle‐shaped morphology with extensive cytoplasmic extensions, consistent with good cell attachment to the scaffold surface. The cells appeared to bridge across pores and interact closely with the scaffold matrix. These qualitative observations suggest improved cell–material interactions; however, no quantitative assessment of cell attachment or spreading was performed.

**Figure 5:**
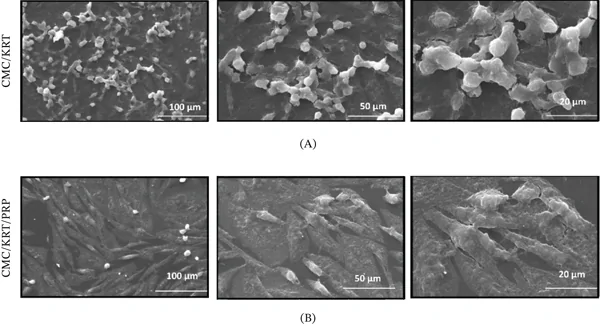
Representative micrographs showing the attachment and spreading of L929 fibroblast cells on (A) CMC/keratin and (B) CMC/keratin/PRF scaffolds after 48 h of culture. Qualitative observations suggested enhanced cell–scaffold interactions in the PRF‐containing composite compared with the CMC/keratin scaffold. The presented SEM images are representative images obtained from three independent experiments.

## 4. Discussion

This study is aimed at investigating the development and performance of a novel CMC/keratin/PRF composite scaffold for periodontal tissue engineering, with emphasis on its structural, mechanical, and biological characteristics. The results collectively demonstrate that the synergistic integration of CMC, keratin, and PRF leads to a bioactive scaffold with properties that make it a promising platform for periodontal tissue engineering.

Macroscopic evaluation confirmed the successful fabrication of self‐supporting and structurally stable scaffolds following freeze‐drying. The preservation of scaffold geometry and the absence of visible defects indicate that the cross‐linked CMC/keratin network provided sufficient mechanical integrity during processing. Similar macroscopic stability has been reported for polysaccharide–protein composite scaffolds designed for tissue engineering applications, where adequate cross‐linking is essential to maintain shape fidelity [35, 36]. Importantly, PRF incorporation did not compromise the gross morphology of the scaffolds, consistent with previous studies demonstrating the feasibility of incorporating PRF into polymeric matrices without impairing structural stability [36, 37]. This feature is particularly relevant for periodontal applications, where scaffolds must withstand handling during surgical implantation.

FTIR studies confirmed effective incorporation and molecular interactions of CMC, keratin, and PRF in the composite scaffold. The existence of typical absorption bands of both polysaccharide and protein components demonstrated the preservation of the major chemical structures of individual constituents after scaffold creation. The absence of a clear Amide III band that is only related to keratin shows the presence of intermolecular interactions, namely, hydrogen bonding between the hydroxyl and carboxyl groups of CMC and the amino groups of keratin. Similar interactions have been discovered for cellulose–protein composite systems and are known to increase structural cohesion and stability of the resultant scaffolds [38, 39]. Amide I band was still identifiable after PRF integration although with a lower intensity. This might be due to overlapping protein signals by keratin and PRF as well as chemical interactions created during GLU cross‐linking. In particular, GLU may react with the amino groups of protein chains to generate Schiff base (C=N) connections, which may cause minor variations in peak intensity and location. Moreover, the presence of an extra absorption band at ∼933 cm^−1^ might be ascribed to the establishment of a more complete cross‐linked network between CMC, keratin, and PRF. Taken together, these spectrum alterations are consistent with effective structural incorporation of all scaffold components. Moreover, the existence of protein‐associated absorption bands after PRF incorporation suggests that the fabrication procedure did not induce considerable chemical alteration of PRF constituents, which is consistent with previous reports on the stability of PRF‐derived biomaterials after scaffold fabrication [36]. In addition, the faint absorption band at about 2361 cm^−1^ is more likely to be attributed to ambient CO_2_ absorbed during FTIR measurement, and not to a particular functional group of the scaffold. The wide absorption band with a maximum at 3200 cm^−1^, due to the overlapping O–H and N–H stretching vibrations, is characteristic of hydrogen‐bonding interactions between CMC and keratin. Decrease in strength or absence of these bands after inclusion of PRF might be due to changes in intermolecular hydrogen‐bonding interactions and spectral overlap after cross‐linking. Overall, FTIR data suggest that the insertion of PRF did not alter the chemical integrity of the scaffold, but instead, it facilitated the construction of a structurally integrated composite network via noncovalent interactions and GLU‐mediated cross‐linking.

SEM study revealed that the microstructure of composite scaffolds was significantly affected by the keratin content. The CMC/keratin (40/60) scaffold showed a more uniform and linked porous structure with larger pore walls than the CMC/keratin (95/5) and CMC/keratin (80/20) formulations, suggesting a better structural organization. Greater keratin amounts may have resulted in improved scaffold integrity due to stronger intermolecular interactions, especially hydrogen bonding, between CMC and keratin at greater keratin concentrations. Improved pore architecture and structural stability have been observed for keratin‐containing composite scaffolds in tissue engineering [40, 41]. The linked porosity network is regarded to be one of the key requirements for tissue engineering applications, as it allows the transport of nutrients and oxygen, elimination of metabolic waste and migration of cells across the scaffold. Therefore, the improved formulation of CMC/keratin (40/60) scaffold was selected for further integration of PRF due to its better pore morphology. The porous architecture of the scaffold was maintained after the introduction of PRF and the scaffold surface was somewhat rougher. This increase in the surface roughness might give extra topographic signals for cell attachment and promote protein adsorption at the surface of the scaffold, thereby resulting in improved cell–material interactions. The inclusion of PRF did not influence the porosity network, indicating that the manufacturing process retained the structural components necessary for tissue regeneration with further biological functioning [42, 43]. In the present work, pore size, porosity, and pore interconnectivity were evaluated subjectively based on SEM observations but not quantitatively quantified. More complete structural characterization might be obtained by future studies employing image analysis or microcomputed tomography (micro‐CT).

The mechanical testing of the PRF‐containing CMC/keratin (40/60) scaffold showed improved mechanical properties. Specifically, the tensile strength, elastic modulus, and toughness of the PRF‐containing scaffold were superior to those of the CMC/keratin scaffold; however, the elongation at break remained almost the same. The observed improvement might be due to the involvement of the fibrin network and proteinaceous constituents of PRF, which can strengthen the scaffold structure and promote stress transmission within the composite matrix. Similar improvements in the mechanical properties of PRF‐containing biomaterials have been documented earlier, where the addition of PRF boosted the stability of the scaffolds without affecting their biocompatibility [38, 44, 45]. The improved toughness also indicates that the composite scaffold would be able to absorb more mechanical energy before failure, which is a desirable feature for periodontal tissue engineering applications since the scaffolds are under mechanical stress under physiological conditions. Overall, the results imply that the introduction of PRF increased the mechanical performance of the optimized scaffold without compromising its structural integrity. It is important to note that the increase in elastic modulus indicates higher stiffness, which is consistent with the observed increase in tensile strength and reflects the reinforcing effect of PRF within the composite matrix.

The MTT assay results showed that all fabricated scaffolds were cytocompatible and supported the viability of L929 fibroblast cells during the culture period. The better cellular response of the CMC/keratin/PRF scaffold could be attributed to the combined biological functions of keratin and PRF. Keratin contains cell‐binding motifs that promote cell attachment, whereas PRF has been widely reported to contain bioactive proteins and growth factors such as PDGF, TGF‐*β*, and VEGF, which were associated with better cellular responses and tissue regeneration in previous studies [32, 46]. However, it is to be highlighted that growth factor release was not explicitly measured in the present investigation and so this process is inferred from prior findings and not experimentally proven [42, 43]. The rise in cell viability from Day 3 to Day 7 shows a steady increment indicating that the scaffold was able to offer a suitable milieu for prolonged cellular activity without any harmful consequences. These findings are in agreement with the previous research revealing an increase in fibroblast proliferation and metabolic activity in PRF‐containing biomaterials. However, only cell viability and metabolic activity were tested using the MTT assay in the present study [46, 47]. The results together suggest that adding PRF to the CMC/keratin scaffold enhances the biological performance of the construct and supports its possible use as a regenerative scaffold for periodontal tissue creation [48, 49].

Qualitative SEM observations suggested improved fibroblast cell contact with the scaffold surface following PRF incorporation. The cells seeded on the CMC/keratin/PRF composite scaffolds appeared to exhibit a well‐spread spindle‐shaped morphology with extended cytoplasmic extensions, suggesting enhanced cell–material interactions compared with the CMC/keratin scaffold. However, it should be emphasized that these findings are based on qualitative assessments and were not confirmed by quantitative analyses such as cell counting, spreading‐area measurement, or morphometric image analysis. Conversely, the generally spherical shape of cells on the CMC/keratin scaffold implies less extent of cytoskeletal organization and focal adhesion production. The improved cell performance on the scaffold with PRF may be due to the synergistic effects of the scaffold composition. Keratin contains bioactive cell‐binding motifs that promote cell attachment. PRF has been shown to include adhesion‐related proteins and growth factors that promote cell attachment, spreading, and proliferation. It should be noted, however, that growth factor release was not directly measured in the present investigation, and the contribution of PRF‐derived growth factors is thus assumed from literature, rather than empirically proven. Moreover, the linked porous architecture along with the enhanced surface roughness seen by the introduction of PRF probably offered additional topographical signals supporting cell anchoring and spreading. These results are in agreement with earlier publications showing enhanced fibroblast adhesion and morphology on PRF‐based composite scaffolds. Collectively, these qualitative observations suggest that the synergistic structural support of CMC, the bioactive properties of keratin, and the biological activity of PRF may provide a favorable microenvironment for cellular interactions, favoring the potential use of the CMC/keratin/PRF scaffold in periodontal tissue engineering [50, 51]. Note that the cell attachment and morphology were evaluated qualitatively based on the SEM observations. The present study did not include quantitative analyses such as cell counting, cell spreading area measurements, or morphometric image analysis, which might further validate these findings in future studies.

Overall, these results provide evidence that the CMC/keratin/PRF composite scaffold combines favorable structural integrity, mechanical performance, and biological activity through synergistic interactions among its components. Compared with previously reported CMC‐ or keratin‐based scaffolds, the incorporation of PRF provides an additional biological advantage by introducing endogenous growth factors that enhance cellular responses. These findings suggest that the proposed composite scaffold is a promising candidate for periodontal tissue engineering, although further studies involving periodontal‐specific cells and in vivo models are required to fully validate its regenerative potential.

## 5. Conclusion

In the present study, a bioactive composite scaffold composed of CMC, keratin, and PRF was successfully fabricated and systematically characterized for periodontal tissue engineering applications. The incorporation of keratin into the CMC matrix significantly improved scaffold morphology and mechanical performance, with the CMC/keratin (40/60) formulation exhibiting the most favorable balance between strength and porosity. The subsequent addition of PRF further enhanced the biological properties of the scaffold without compromising its structural integrity. The CMC/keratin/PRF composite scaffold demonstrated excellent cytocompatibility and promoted higher cell viability compared with scaffolds without PRF, as determined by the MTT assay. Qualitative SEM observations also suggested improved cell adhesion and spreading on the PRF‐containing composite scaffold. These improvements may be associated with the presence of bioactive cell‐binding motifs in keratin together with the well‐documented biological activity of PRF. Nevertheless, growth factor release was not directly investigated in this study and should be evaluated in future work. Although the current study is limited to in vitro evaluations using a fibroblast cell model, the obtained results highlight the potential of the CMC/keratin/PRF scaffold as a bioactive platform for periodontal tissue engineering. Future studies should focus on investigating its performance with periodontal‐specific cell types and in vivo models to further assess its clinical applicability and regenerative efficacy.

## Author Contributions

Saba Jaffarkhah contributed to the experimental work and manuscript preparation. Raziyeh Najafloo participated in data acquisition and methodology development. Saeedeh Zare Jalise performed scaffold fabrication and characterization experiments. Mina Jarrahi assisted with data analysis and interpretation of the results. Esmaeil Biazar supervised the study and critically revised the manuscript. Arezou Mehrabi was the corresponding author, conceived and designed the study, and finalized the manuscript.

## Funding

No funding was received for this manuscript.

## Disclosure

All authors read and approved of the final manuscript.

## Ethics Statement

The study involving human participants was approved by the Institutional Research Ethics Committee (Approval No: IR.IAU.TON.REC.1404.152).

## Consent

Written informed consent was obtained from all participants prior to blood collection.

## Conflicts of Interest

The authors declare no conflicts of interest.

## Data Availability

The data that support the findings of this study are available from the corresponding author upon reasonable request.

## References

[1] Könönen E. , Gursoy M. , and Gursoy U. K. , Periodontitis: A Multifaceted Disease of Tooth-Supporting Tissues, Journal of Clinical Medicine. (2019) 8, no. 8, 10.3390/jcm8081135, 31370168. PMC6723779 31370168

[2] Williams R. C. , Periodontal Disease, New England Journal of Medicine. (1990) 322, no. 6, 373–382, 10.1056/NEJM199002083220606. 2405268

[3] Niemiec B. A. , Periodontal Disease, Topics in Companion Animal Medicine. (2008) 23, no. 2, 72–80, 10.1053/j.tcam.2008.02.003. 18482707

[4] Chen F.-M. and Jin Y. , Periodontal Tissue Engineering and Regeneration: Current Approaches and Expanding Opportunities, Tissue Engineering Part B: Reviews. (2010) 16, no. 2, 219–255, 10.1089/ten.teb.2009.0562, 19860551. 19860551

[5] Deas D. E. , Moritz A. J. , Sagun R. S. , Gruwell S. F. , and Powell C. A. , Scaling and Root Planing vs. Conservative Surgery in the Treatment of Chronic Periodontitis, Periodontology. (2016) 71, no. 1, 128–139, 10.1111/prd.12114. 27045434

[6] Cho Y.-D. , Kim K. H. , Lee Y. M. , Ku Y. , and Seol Y. J. , Periodontal Wound Healing and Tissue Regeneration: A Narrative Review, Pharmaceuticals. (2021) 14, no. 5, 10.3390/ph14050456, 34065862. PMC8151433 34065862

[7] Duarte N. D. , Frigério P. B. , Chica G. E. A. , Okamoto R. , Buchaim R. L. , Buchaim D. V. , Messora M. R. , and Issa J. P. M. , Biomaterials for Guided Tissue Regeneration and Guided Bone Regeneration: A Review, Dentistry Journal. (2025) 13, no. 4, 10.3390/dj13040179, 40277509. PMC12026320 40277509

[8] Soldatos N. K. , Stylianou P. , Koidou V. P. , Angelov N. , Yukna R. , and Romanos G. E. , Limitations and Options Using Resorbable Versus Nonresorbable Membranes for Successful Guided Bone Regeneration, Quintessence International. (2017) 48, no. 2, 131–147, 10.3290/j.qi.a37133, 27834419. 27834419

[9] Liu Y. , Guo L. , Li X. , Liu S. , du J. , Xu J. , Hu J. , and Liu Y. , Challenges and Tissue Engineering Strategies of Periodontal-Guided Tissue Regeneration, Tissue Engineering Part C: Methods. (2022) 28, no. 8, 405–419, 10.1089/ten.tec.2022.0106. 35838120

[10] Chen H. , Song G. , Xu T. , Meng C. , Zhang Y. , Xin T. , Yu T. , Lin Y. , and Han B. , Biomaterial Scaffolds for Periodontal Tissue Engineering, Journal of Functional Biomaterials. (2024) 15, no. 8, 10.3390/jfb15080233, 39194671. PMC11355167 39194671

[11] Vaquette C. , Pilipchuk S. P. , Bartold P. M. , Hutmacher D. W. , Giannobile W. V. , and Ivanovski S. , Tissue Engineered Constructs for Periodontal Regeneration: Current Status and Future Perspectives, Advanced Healthcare Materials. (2018) 7, no. 21, e1800457, 10.1002/adhm.201800457, 30146758. 30146758

[12] Farahani M. , Gnatowski P. , Najafloo R. , Navaei T. , Piłat E. , Kucińska-Lipka J. , Saeb M. R. , and Mozafari M. , Enhancing Diaphragmatic Defect Repair and Regeneration: How Biomaterials Leading the Way to Progress?, Biomedical Engineering Materials Journal. (2024) 2, no. 3, e12070, 10.1002/bmm2.12070.

[13] Milan P. B. , Amini N. , Mehrabi A. , Mousazadeh S. , Ababzadeh S. , and Rezapour A. , Cell Sources in Cardiac Tissue Engineering: Current Choices, Current Stem Cell Research & Therapy. (2021) 16, no. 6, 745–752, 10.2174/1574888X16666210120101223, 33494682. 33494682

[14] Angjelova A. , Jovanova E. , Polizzi A. , Annunziata M. , Laganà L. , Santonocito S. , and Isola G. , Insights and Advancements in Periodontal Tissue Engineering and Bone Regeneration, Medicina. (2024) 60, no. 5, 10.3390/medicina60050773, 38792956. PMC11123221 38792956

[15] Rahman M. S. , Hasan M. S. , Nitai A. S. , Nam S. , Karmakar A. K. , Ahsan M. S. , Shiddiky M. J. A. , and Ahmed M. B. , Recent Developments of Carboxymethyl Cellulose, Polymers. (2021) 13, no. 8, 10.3390/polym13081345. PMC8074295 33924089

[16] Pezeshkian Z. , Peyravian N. , Najafloo R. , MalekzadehKebria M. , Hadi M. , Valizadeh H. , Sarmadi V. H. , and Milan P. B. , Insights Into Cellulose Nanocrystals-Based Bioinks for 3D Bioprinting in Regenerative Medicine and Tissue Engineering: A Review, International Journal of Biological Macromolecules. (2026) 337, 149409, 10.1016/j.ijbiomac.2025.149409. 41344460

[17] Liuyun J. , Yubao L. , and Chengdong X. , Preparation and Biological Properties of a Novel Composite Scaffold of Nano-Hydroxyapatite/Chitosan/Carboxymethyl Cellulose for Bone Tissue Engineering, Journal of Biomedical Science. (2009) 16, no. 1, 10.1186/1423-0127-16-65, 19594953. PMC2720940 19594953

[18] Huang W.-H. , Hung C. Y. , Chiang P. C. , Lee H. , Lin I. T. , Lai P. C. , Chan Y. H. , and Feng S. W. , Physicochemical Characterization, Biocompatibility, and Antibacterial Properties of CMC/PVA/Calendula officinalis Films for Biomedical Applications, Polymers. (2023) 15, no. 6, 10.3390/polym15061454, 36987233. PMC10059992 36987233

[19] Mousazadeh S. , Mehrabi A. , Mollafilabi A. , Sherafati S. , and Kiani J. , Bioengineered 3D Porous Silk Fibroin/Decellularized Human Placenta Incorporating Elastin-Like Polypeptides (ELP) Scaffold for Accelerated Wound Healing: In-Vitro and In-Vivo Studies, Journal of Drug Delivery Science and Technology. (2025) 113, 107351, 10.1016/j.jddst.2025.107351.

[20] Mehrabi A. , Jalise S. Z. , Hivechi A. , Habibi S. , Kebria M. M. , Haramshahi M. A. , Latifi N. , Karimi A. , and Milan P. B. , Evaluation of Inherent Properties of the Carboxymethyl Cellulose (CMC) for Potential Application in Tissue Engineering Focusing on Bone Regeneration, Polymers for Advanced Technologies. (2024) 35, no. 1, e6258, 10.1002/pat.6258.

[21] Salleh K. M. and Abd N. F. , Rashid, Keratin-Based Biomaterials for Biomedical Applications, Polymer Composites Derived From Animal Sources, 2024, Elsevier, 219–242, 10.1016/B978-0-443-22414-0.00012-0.

[22] Tan B. Y. , Understanding of 2D Human Hair Keratin Platforms for Cell Culture Applications, 2020, Nanyang Technological University.

[23] Soleymani Eil Bakhtiari S. and Karbasi S. , Keratin-Containing Scaffolds for Tissue Engineering Applications: A Review, Journal of Biomaterials Science, Polymer Edition. (2024) 35, no. 6, 916–965, 10.1080/09205063.2024.2311450, 38349200. 38349200

[24] Tollabi M. , Mehrabi A. , Zolfaghari S. , Khalili M. R. , Shokrgozar M. A. , and Milan P. B. , Biomaterial-Based Cell Membrane Engineering Strategies and Their Scope in Biomedical Applications, Cell Membrane Engineering for Advancing Cell-Based and Nanotechnology-Based Therapies, 2025, Springer, 185–215, 10.1007/978-3-031-93425-4_7.

[25] Mehrabi A. , Najafloo R. , Valizadeh H. , Sarmadi V. H. , Naderi S. , Faghihebadi G. , and Milan P. B. , Natural Polymers for Diagnosis and Treatment of Cancers, Biomaterials for Precision Cancer Medicine, 2025, Elsevier, 123–153, 10.1016/B978-0-323-85661-4.00017-2.

[26] Lee S.-H. and Shin H. , Matrices and Scaffolds for Delivery of Bioactive Molecules in Bone and Cartilage Tissue Engineering, Advanced Drug Delivery Reviews. (2007) 59, no. 4-5, 339–359, 10.1016/j.addr.2007.03.016. 17499384

[27] Najafloo R. , Milan P. B. , Karimi A. , Bagher Z. , Kalmer R. R. , Ghasemian M. , and Faridi-Majidi R. , Crosslinking Gelatin With Robust Inherent Antibacterial Natural Polymer for Wound Healing, International Journal of Biological Macromolecules. (2024) 280, Part 4, 136144, 10.1016/j.ijbiomac.2024.136144, 39353527. 39353527

[28] Caruana A. , Savina D. , Macedo J. P. , and Soares S. C. , From Platelet-Rich Plasma to Advanced Platelet-Rich Fibrin: Biological Achievements and Clinical Advances in Modern Surgery, European Journal of Dentistry. (2019) 13, no. 2, 280–286, 10.1055/s-0039-1696585. PMC6777161 31509878

[29] Saglanmak A. , Cinar C. , Gultekin A. , Saglanmak A. , Cinar C. , and Gultekin A. , Platelet Rich Fibrin (PRF) Application in Oral Surgery, Platelets. (2020) 1, 1–14.

[30] Tollabi M. , Kheirandish M. H. , Mehrabi A. , Poursalehi Z. , Heidari M. , and Milan P. B. , Genetically Mediated Cell Membrane Engineering Strategies and Their Scope in Biomedical Applications, Cell Membrane Engineering for Advancing Cell-Based and Nanotechnology-Based Therapies, 2025, Springer, 153–183, 10.1007/978-3-031-93425-4_6.

[31] Bielecki T. and Dohan Ehrenfest D. M. , Platelet-Rich Plasma (PRP) and Platelet-Rich Fibrin (PRF): Surgical Adjuvants, Preparations for In Situ Regenerative Medicine and Tools for Tissue Engineering, Current Pharmaceutical Biotechnology. (2012) 13, no. 7, 1121–1130, 10.2174/138920112800624292, 21740380. 21740380

[32] Farshidfar N. , Ahmad P. , Estrin N. , Zhang Y. , and Miron R. J. , Use of Platelet-Rich Fibrin (PRF) on Palatal Wound Healing: A Narrative Review With Clinical Recommendations, Journal of Periodontal Research, 2025, Wiley Online Library, 10.1111/jre.13413, 40492943. 40492943

[33] Bıçakçıoğlu H. A. and Çolak G. , Platelet-Rich Fibrin-Derived Extracellular Vesicles: Emerging Biological Mediators in Periodontal Regeneration, Odontology. (2026) 114, no. 3, 1086–1098, 10.1007/s10266-025-01275-2, 41350499. 41350499

[34] Najafloo R. , Imani R. , Behyari M. , and Nour S. , Synthesis and Characterization of Thymol-Loaded Niosomal Film for the Prevention of Implant-Related Infection, Iranian Biomedical Journal. (2023) 27, no. 2, 117–125, 10.61186/ibj.3788. PMC10314763 37070674

[35] Iravani S. and Varma R. S. , Cellulose-Based Composites as Scaffolds for Tissue Engineering: Recent Advances, Molecules. (2022) 27, no. 24, 10.3390/molecules27248830, 36557963. PMC9784432 36557963

[36] Karimi F. , Biazar E. , Heidari-Keshel S. , Pourjabbar B. , Khataminezhad M. R. , Shirinbakhsh S. , and Zolfaghari-Moghaddam S. Y. , Platelet-Rich Fibrin (PRF) Gel Modified by a Carbodiimide Crosslinker for Tissue Regeneration, RSC Advances. (2022) 12, no. 21, 13472–13479, 10.1039/D2RA00985D, 35527730. PMC9069288 35527730

[37] Ieviņa L. and Dubņika A. , Navigating the Combinations of Platelet-Rich Fibrin With Biomaterials Used in Maxillofacial Surgery, Frontiers in Bioengineering and Biotechnology. (2024) 12, 1465019, 10.3389/fbioe.2024.1465019, 39434715. PMC11491360 39434715

[38] Krishani M. , Chong J. N. , Lim W. R. , Jusoh N. , Sambudi N. S. , and Suhaimi H. , Synthesis and Characterization of Keratin-Based Scaffold for Potential Tissue Engineering Applications, Fibers. (2025) 13, no. 7, 10.3390/fib13070097.

[39] Kakkar P. , Verma S. , Manjubala I. , and Madhan B. , Development of Keratin–Chitosan–Gelatin Composite Scaffold for Soft Tissue Engineering, Materials Science and Engineering: C. (2014) 45, 343–347, 10.1016/j.msec.2014.09.021, 25491838. 25491838

[40] Puglia D. , Ceccolini R. , Fortunati E. , Armentano I. , Morena F. , Martino S. , Aluigi A. , Torre L. , and Kenny J. M. , Effect of Processing Techniques on the 3Dmicrostructure of Poly (l-Lactic Acid) Scaffolds Reinforced With Wool Keratin From Different Sources, Journal of Applied Polymer Science. (2015) 132, no. 48, 10.1002/app.42890.

[41] Freigassner J. , van Nieuwenhoven R. W. , and Gebeshuber I. C. , From Nanostructure to Function: Hierarchical Functional Structures in Chitin and Keratin, Zeitschrift für Physikalische Chemie. (2025) 239, no. 9, 1443–1497, 10.1515/zpch-2024-0913.

[42] Wang X. , Zhang Y. , Choukroun J. , Ghanaati S. , and Miron R. , Behavior of Gingival Fibroblasts on Titanium Implant Surfaces in Combination With Either Injectable-PRF or PRP, International journal of Molecular Sciences. (2017) 18, no. 2, 10.3390/ijms18020331. PMC5343867 28165420

[43] Lollobrigida M. , Maritato M. , Bozzuto G. , Formisano G. , Molinari A. , and de Biase A. , Biomimetic Implant Surface Functionalization With Liquid L-PRF Products: In Vitro Study, BioMed Research International. (2018) 2018, no. 1, 9031435, 10.1155/2018/9031435. PMC5964419 29854805

[44] Simões-Pedro M. , Tróia P. M. B. P. S. , dos Santos N. B. M. , Completo A. M. G. , Castilho R. M. , and de Oliveira Fernandes G. V. , Tensile Strength Essay Comparing Three Different Platelet-Rich Fibrin Membranes (L-PRF, A-PRF, and A-PRF+): A Mechanical and Structural In Vitro Evaluation, Polymers. (2022) 14, no. 7, 10.3390/polym14071392, 35406263. PMC9002533 35406263

[45] Mirhaj M. , Salehi S. , Tavakoli M. , Varshosaz J. , Labbaf S. , Abadi S. A. M. , and Haghighi V. , Comparison of Physical, Mechanical and Biological Effects of Leucocyte-PRF and Advanced-PRF on Polyacrylamide Nanofiber Wound Dressings: In Vitro and In Vivo Evaluations, Biomaterials Advances. (2022) 141, 213082, 10.1016/j.bioadv.2022.213082, 36067641. 36067641

[46] Feroz S. , Muhammad N. , Ullah R. , Nishan U. , Cathro P. , and Dias G. , Mechanical Properties, and In Vitro Biocompatibility Assessment of Biomimetic Dual Layered Keratin/Hydroxyapatite Scaffolds, Frontiers in Bioengineering and Biotechnology. (2023) 11, 1304147, 10.3389/fbioe.2023.1304147, 38173873. PMC10764155 38173873

[47] Carvalho C. R. , Costa J. B. , Costa L. , Silva-Correia J. , Moay Z. K. , Ng K. W. , Reis R. L. , and Oliveira J. M. , Enhanced Performance of Chitosan/Keratin Membranes With Potential Application in Peripheral Nerve Repair, Biomaterials Science. (2019) 7, no. 12, 5451–5466, 10.1039/C9BM01098J, 31642822. 31642822

[48] Xue P.-P. , Yuan J. D. , Yao Q. , Zhao Y. Z. , and Xu H. L. , Bioactive Factors-Imprinted Scaffold Vehicles for Promoting Bone Healing: The Potential Strategies and the Confronted Challenges for Clinical Production, BIO Integration. (2020) 1, no. 1, 37–54, 10.15212/bioi-2020-0010.

[49] Klimek K. and Ginalska G. , Proteins and Peptides as Important Modifiers of the Polymer Scaffolds for Tissue Engineering Applications—A Review, Polymers. (2020) 12, no. 4, 10.3390/polym12040844, 32268607. PMC7240665 32268607

[50] Kasnak G. , Fteita D. , Jaatinen O. , Könönen E. , Tunali M. , Gürsoy M. , and Gürsoy U. K. , Regulatory Effects of PRF and Titanium Surfaces on Cellular Adhesion, Spread, and Cytokine Expressions of Gingival Keratinocytes, Histochemistry and Cell Biology. (2019) 152, no. 1, 63–73, 10.1007/s00418-019-01774-8. 30767049

[51] Strauss F.-J. , Nasirzade J. , Kargarpoor Z. , Stähli A. , and Gruber R. , Effect of Platelet-Rich Fibrin on Cell Proliferation, Migration, Differentiation, Inflammation, and Osteoclastogenesis: A Systematic Review of In Vitro Studies, Clinical Oral Investigations. (2020) 24, no. 2, 569–584, 10.1007/s00784-019-03156-9, 31879804. PMC6988133 31879804

